# Physical activity patterns and the risk of colorectal cancer in the Norwegian Women and Cancer study: a population-based prospective study

**DOI:** 10.1186/s12885-018-5092-0

**Published:** 2018-12-04

**Authors:** Sunday Oluwafemi Oyeyemi, Tonje Braaten, Idlir Licaj, Eiliv Lund, Kristin Benjaminsen Borch

**Affiliations:** 1 Department of Community Medicine, Faculty of Health Sciences, UiT-The Arctic University of Norway, Tromsø, Norway; 2 Clinical Research Department, Centre François Baclesse, Normandie University, UNICAEN, INSERM, U1086, Caen, France

**Keywords:** Physical activity, Colon cancer, Rectal cancer, Colorectal cancer, Women, NOWAC

## Abstract

**Introduction:**

Colorectal cancer (CRC) remains the second most common cancer in women worldwide. Physical activity (PA) has been associated with reduced risk of CRC; however, this has been demonstrated more consistently in men, while results of studies in women have been largely equivocal. We aimed to further examine the relationship between PA patterns and the risk of CRC in women, using repeated measurements.

**Methods:**

We followed participants of the Norwegian Women and Cancer (NOWAC) Study - a nationally representative cohort. Baseline information was available for 79,184 women, and we used this information in addition to follow-up information collected 6–8 years later, for repeated measurement analysis. At enrollment, participants were cancer-free and aged 30–70 years, with a median age of 51 years. We used Cox proportional hazards regression to compute hazard ratios (HRs) and 95% confidence intervals (CIs).

**Results:**

During an average of 14.6 years of follow-up and 1.16 million person-years, 885 cases of colon and 426 cases of rectal cancer were identified through linkage to the Norwegian Cancer Registry (median age at diagnosis: 65 years). We found no association between PA level and the risk of colon cancer in baseline or repeated measurements analyses when comparing women with PA level 1–2 to those with PA level 5–6 (reference) (baseline: HR = 0.90, 95% CI 0.66–1.23, *p*-trend = 0.76; repeated measurements: HR = 0.78, 95% CI 0.55–1.10, *p*-trend = 0.27). Results were the same when comparing PA level 9–10 to the reference level (baseline: HR = 0.80, 95% CI 0.56–1.12, *p*-trend = 0.76; repeated measurements: HR = 0.82, 95% CI 0.58–1.16, *p*-trend = 0.27). Similarly, we found no association between PA levels and the risk of rectal cancer.

**Conclusions:**

Women may need to look beyond PA in order to reduce their risk of CRC.

## Background

Colorectal cancer (CRC) remains the second most common cancer in women worldwide [1]. This is also true in Norway, where CRC is the second most common cancer in women [2]. In 2018, it was estimated that Norway had the highest incident rate of CRC in women worldwide, at 39.3 per 100,000, compared to 24.2 per 100,000 in the rest of Europe (World age-standardised rate) [1, 3]. The average annual number of new cases in women in Norway has been on the increase in the past few years, with 1706 in 2002–06; 1833 in 2007–11; and 2049 in 2012–16 [2].

There is convincing epidemiological evidence suggesting that a healthy lifestyle, body weight, and diet could substantially prevent the development of CRC [4], and several epidemiological studies have demonstrated a risk-reducing association between physical activity (PA) and CRC [5–8]. The Continuous Update Project on colorectal cancer by the World Cancer Research Fund/American Institute for Cancer Research (WCRF/AICR) published in September 2017 concluded that all domains of PA (occupational, household, transport, and recreational) reduce the risk of CRC [9]. However, this has only been demonstrated consistently in men, while results of such studies in women have been largely equivocal [10, 11]. Considering only prospective studies that either included women alone or presented sex-specific findings, 13 studies reported no associations between PA and CRC among women with relative risks ranging from 0.69 to 1.15 [10–22]. Six studies reported statistically significant inverse associations among women with relative risks ranging from 0.54 to 0.90 [6–8, 23–25], which were consistent with the findings of most studies in men. However, the associations in women were weaker than those in men, and some of the significant observations in women were only present in sub-analyses [11, 26].

These discrepancies may have stemmed from methodological differences, such as relatively small sample sizes, deficient or poor assessment methods for PA, or assessment of different domains of PA by methods of unknown validity or reproducibility. It may be that the assessment of PA in women has more intricacies than that in men, as inclusion of household PA in women may be under- (or over-) rated [27]. It is also plausible that a sex difference exists in the physio-biological response to PA [28, 29].

The aim of the present study was to further examine the relationship between PA patterns and the risk of CRC in women, using a validated, single-item, self-administered questionnaire and repeated measurements, in a nationally representative cohort of Norwegian women.

## Methods

### The Norwegian women and Cancer study

The Norwegian Women and Cancer (NOWAC) Study is a nationally representative, prospective cohort study which started in 1991. The details of the cohort are fully described elsewhere [30, 31]. In summary, invitations to participate in the NOWAC Study were sent to a sample of women aged 30–70 years, who were randomly selected from the Norwegian Central Population Register. The participants were recruited in three waves: 1991–92, 1996–97, and 2003–04. More than 172,000 women agreed to participate and completed questionnaires regarding their lifestyle and health status. All participating women gave written informed consent, and the overall response rate was 52.7%. The NOWAC Study was approved by the Regional Committee for Medical Research Ethics and the Norwegian Data Inspectorate.

### Study sample

**Fig. 1:**
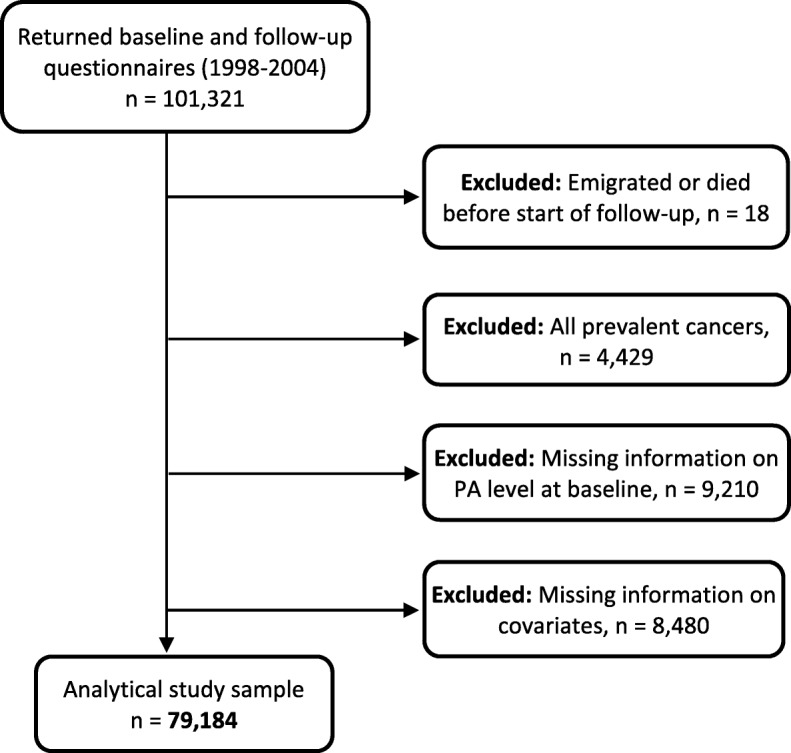
Flowchart for study sample

In these analyses, we used information from 101,321 women who were recruited in 1991–92, 1996–97, and 2003–04, and completed food frequency questionnaires in 1998, 1996–97 and 2003–04, respectively (baseline); and follow-up questionnaires 6–8 years after baseline questionnaire (repeated measurement). We excluded women who emigrated or died before the start of follow-up (*n* = 18), those with prevalent cancer other than non-melanoma skin cancer at baseline (*n* = 4429), those with missing information on PA level at baseline (*n* = 9210), and those with missing information on any of the covariates at baseline (height and weight (used to calculate body mass index), duration of education, alcohol consumption, smoking status, and intake of red meat, processed meat, dietary calcium and dietary fibre) (*n* = 8480). Thus the final analytical sample consisted of 79,184 women (Fig. 1). In the repeated measurement analysis, we used measurements from baseline (first measurements) and follow-up information (second measurements) of PA, BMI, and smoking status. Thereafter follow-up information was applied until emigration, death, cancer diagnosis, or the end of the study period, whichever occurred first.

**Fig. 2:**
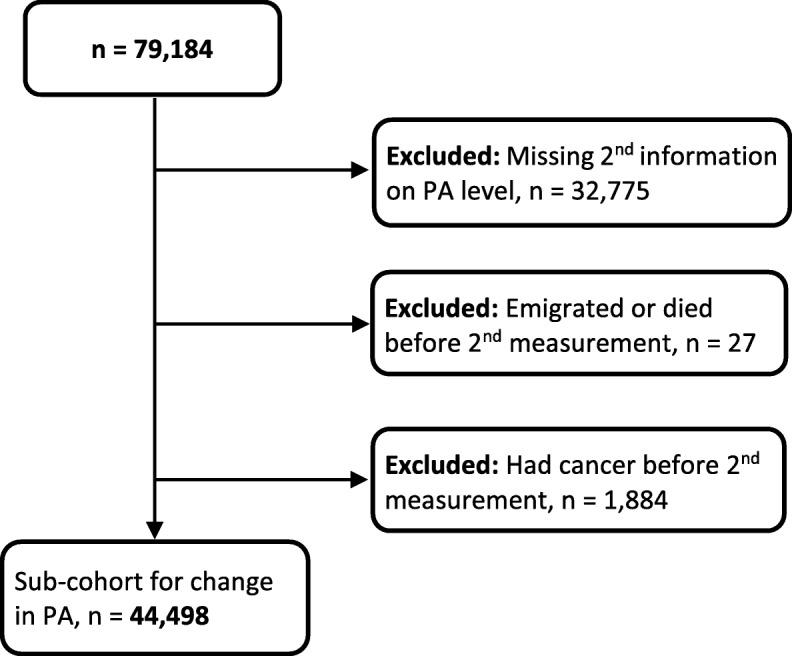
Flowchart for sub-cohort (used for additional analyses of change in PA)

We also carried out separate analyses where we used change in PA level between baseline and follow-up as the exposure variable. These analyses consisted of 44,498 women who had both baseline and follow-up information on PA level, after exclusion of those who died (*n* = 3), emigrated (*n* = 24), or had cancer (*n* = 1884) before the follow-up measurement took place (Fig. 2).

### Assessment of physical activity level and covariates

Information on PA level was taken from the NOWAC questionnaires. The baseline and follow-up questionnaires contained the same question on PA level. The participants were asked, “*By physical activity we mean activity both at work and outside work, at home, as well as training/exercise and other physical activity, such as walking, etc. Please mark the number that best describes your level of physical activity; 1 being very low and 10 being very high*”.

The PA scale used in this study reflects the *total* amount of PA, which includes the domains (occupational, household, transport, and recreational), in one global score. This PA scale has been validated to rank PA levels in the Norwegian female population, and a moderate, but significant Spearman’s rank correlation coefficient was found (range: 0.36–0.46; *p* < 0.001) between the PA scale and the outcomes from the measurements of a combined sensor monitoring heart rate and movement [32].

Information on initial covariates obtained through the NOWAC questionnaires at baseline included age, height, BMI, duration of education, household income, alcohol consumption, smoking status, use of hormone replacement therapy, intake of red meat, processed meat, dietary calcium, and dietary fibre. The choice of these covariates was based on documented risk factors in the literature and in previous similar studies [10–12, 26].

### Cancer incidence, emigration, and death

NOWAC participants diagnosed with primary CRC using the International Statistical Classification of Diseases and Related Health Problems, Tenth Edition (ICD-10 code C18 or C19–20), were identified through linkage to the Cancer Registry of Norway with the aid of the unique national identity number. The Cancer Registry of Norway has been judged to be more than 98% complete [33]. Information on date of emigration and death in the cohort was obtained through linkage to the Norwegian Central Population Register.

### Statistical methods

#### Analyses using baseline data

We used Cox proportional hazards models, with age as the time scale, to estimate hazard ratios (HRs) with 95% confidence intervals (CIs) for the associations between PA levels and risk of CRC. PA levels at baseline were divided into five groups [1–10], was used as the reference group. We used similar models to estimate multivariable-adjusted HRs with 95% CIs. We stratified all the models by recruitment sub-cohort (1991–92, 1996–97, and 2003–04) to control for potential differences in the three recruitment waves. In the Cox models, follow-up time was defined as the interval between age at baseline and age at emigration, death, diagnosis of any incident cancer, or age at the end of the study period (31 December 2015), whichever occurred first.

We checked the proportional hazards assumption by testing an interaction variable between the groups of PA levels and the logarithm of the age of the participants. We carried out an initial analysis on the baseline data to select the covariates to adjust for in the final models. This initial analysis included: height (continuous, in metres); body mass index calculated from weight divided by the square of the height (BMI, < 25.0, 25.0–29.9, ≥30.0 kg/m^2^); duration of education (< 10, 10–12, ≥13 years, corresponding to primary and lower secondary school, upper secondary school, and higher education, respectively); household income (< 300,000; 300,000-600,000; > 600,000 Norwegian krone per annum, corresponding to low, medium and high income); alcohol consumption (0, ≤3, > 3 g/day); smoking status (never, former, current); hormone replacement therapy (never, former, current); red meat intake (0, ≤15, > 15 g/day); processed meat intake (0, ≤30, > 30 g/day); dietary calcium (< 700, ≥700 mg/day) and dietary fibre (≤21, > 21 g/day). Only covariates associated with a change of at least 10% in the regression coefficient of any of the groups of the PA levels were included in final models. All the above covariates met this criterion except hormone replacement therapy, household income, and red meat intake. However, the latter was still added to the models because of its reported association in the carcinogenesis of colorectal tissues [34].

We assessed possible interactions between PA and BMI, duration of education, alcohol consumption, and smoking status, respectively. We further explored the relationship between PA levels and CRC stratified by BMI categories, as obesity has been deemed as a convincing factor in the development of CRC [35, 36]. We tested for linear trend by using the original 10-level PA scale modelled as a continuous variable. We conducted sensitivity analyses by re-categorising the PA levels into three groups [1–10], and using the baseline information. We also repeated baseline analyses after excluding cancers diagnosed during the first 2 years of the follow-up in order to control for possible reverse causality.

#### Analyses using repeated measurements of physical activity level

We used baseline information on PA level until follow-up information became available. Subsequently, we applied follow-up information until emigration, death, cancer diagnosis, or the end of the study period (31 December 2015), whichever came first. Follow-up information on BMI and smoking status was also applied once available.

#### Analyses according to change in physical activity level

We grouped the 10 PA levels into three categories at baseline: ‘inactive’, (PA level 1–4), ‘moderately active’ (PA level 5–6), and ‘active’ (PA level 7–10). We then used the follow-up data on PA level to categorize participants as ‘consistently active’ (PA level 7–10 at baseline and follow-up), ‘consistently moderately active’ (PA level 5–6 at baseline and follow-up), ‘consistently inactive’ (PA level 1–4 at baseline and follow-up), ‘increased PA’ (increased PA level between baseline and follow-up), and ‘decreased PA’ (decreased PA level between baseline and follow-up).

We then used this change in PA level as the exposure variable and adjusted for the time period between the two measurements. Thus, we considered participants to be at risk from the date of the follow-up measurement until emigration, death, CRC diagnosis, or the end of the study period (31 December 2015), whichever came first.

All statistical tests were two-sided, and all statistical analyses were conducted using Stata for Windows version 15.0 (StataCorp, College Station, Texas, USA). All *p* values were considered statistically significant at a level of < 0.05.

## Results

During an average of 14.6 years of follow-up and 1.16 million person-years, 885 cases of colon cancer and 426 cases of rectal cancer were diagnosed. The median age of the cohort at baseline was 51 years, while the median age at diagnosis was 65 years, ranging from 43 to 87 years.

At baseline, 43% of the cohort reported PA levels 5–6, and 74% reported a PA level of 5 or higher (Table 1). Compared to participants with PA levels 1–4, women with PA levels 5–10 had a lower mean BMI (24.3 vs 26.0 kg/m^2^), similar mean age (51.3 vs 52.2 years), similar mean duration of education (12.4 vs 12.0 years), and same daily alcohol consumption (3.5 vs 3.5 g/day). Furthermore, women with PA levels 5–10 were more often never smokers (38% vs 36%), less often current smokers (29% vs 33%), consumed slightly less red meat (15.3 vs 16.0 g/day), less processed meat (33.3 vs 34.8 g/day), more dietary calcium (763 vs 717 mg/day), and more dietary fibre (22.0 vs 20.0 g/day), than women with PA levels 1–4.

**Table 1:** Characteristics of participants in NOWAC Study by physical activity level at baseline (*n* = 79,184)

Characteristics	Physical activity level at baseline				
	1–2	3–4	5–6	7–8	9–10
Study population (N = 79,184)	3616 (4.6%)	17,360 (21.9%)	34,208 (43.2%)	20,029 (25.3%)	3971 (5.0%)
Mean age (±SE)	52.91 (0.11)	52.00 (0.05)	51.38 (0.03)	51.11 (0.04)	51.65 (0.10)
Person-years at risk ^a^	51,685	255,773	503,850	289,189	57,422
Average follow-up time (SD) ^b^	14.29 years (4.62)	14.73 years (4.10)	14.73 year (3.93)	14.44 years (3.88)	14.46 years (3.92)
Colon cancer (885)	45 (1.25%)	203 (1.17%)	393 (1.15%)	208 (1.04%)	36 (0.91%)
Rectal cancer (426)	24 (0.66%)	100 (0.58%)	175 (0.51%)	99 (0.49%)	28 (0.71%)
Colorectal cancer (1311)	69 (1.91%)	303 (1.75%)	568 (1.66%)	307 (1.53%)	64 (1.61%)
Mean height in *cm* (±SE)	165.9 (0.10)	166.1 (0.04)	166.3 (0.03)	166.5 (0.04)	166.1 (0.09)
Mean BMI in *kg/m*^*2*^ (±SE)	26.92 (0.09)	25.81 (0.03)	24.61 (0.02)	23.83 (0.02)	23.58 (0.05)
Mean duration of education in *years* (±SE)	11.46 (0.06)	12.15 (0.03)	12.28 (0.02)	12.62 (0.03)	11.85 (0.06)
Mean alcohol consumption, *grams* (±SE)	3.25 (0.08)	3.51 (0.03)	3.48 (0.02)	3.64 (0.03)	3.25 (0.07)
Smoking status, *n (%)*					
Never 29,292 *(37.0%)*	1081 (29.9%)	6370 (36.7%)	12,871 (37.6%)	7564 (37.8%)	1406 (35.4%)
Former 26,387 *(33.3%)*	1104 (30.5%)	5475 (31.5%)	11,335 (33.1%)	7128 (35.6%)	1345 (33.9%)
Current 23,505 *(29.7%)*	1431 (39.6%)	5515 (31.8%)	10,002 (29.2%)	5337 (26.7%)	1220 (30.7%)
Mean daily intakes in *grams*					
Red meat (±SE)	16.45 (0.22)	15.86 (0.09)	15.31 (0.06)	15.19 (0.08)	15.39 (0.20)
Processed meat (±SE)	35.57 (0.41)	34.69 (0.17)	33.92 (0.12)	32.44 (0.15)	32.72 (0.38)
Dietary calcium (±SE)	698.16 (5.46)	720.64 (2.29)	748.91 (1.64)	779.19 (2.24)	807.32 (5.67)
Dietary fibre (±SE)	18.85 (0.12)	20.20 (0.05)	21.45 (0.04)	22.58 (0.05)	23.53 (0.12)

^a^Total person years = 1,157,919

^b^Average follow-up time = 14.62 years (SD = 3.99, SE = 0.01)

In the multivariable baseline analyses, we found no statistical significant association between PA level and the risk of CRC when women with PA level 9–10 were compared to those with PA level 5–6 (colon: HR = 0.80, 95% CI 0.56–1.12, *p*-trend = 0.76; rectal: HR = 1.40, 95% CI 0.94–2.10, *p*-trend = 0.87) (Table 2). This null relationship did not change after excluding those who were diagnosed with cancer in the first 2 years of follow-up (*data not shown*). We explored the outcome of re-categorising the PA levels into three groups: 1–4, 5–6, and 7–10, with 5–6 as the reference group and using the baseline information. This does not change the effects, *p*-trend nor the overall findings (*data not shown*). Furthermore, interaction terms between PA levels and categories of BMI, duration of education, alcohol consumption, and smoking status were not significant. In analyses stratified by BMI, we found no association between PA level and CRC (*data not shown*).

**Table 2:** Hazard ratios (95% CI) of colon, rectal, and colorectal cancers by physical activity level at baseline (*n* = 79,184) in the NOWAC Study

		Physical activity level at baseline					
Cancer	Models	1–2	3–4	5–6	7–8	9–10	*p* trend
Colon	Age-adjusted *N of cases: 885*	0.98 (0.72–1.33) *45*	0.94 (0.80–1.12) *203*	1.00 *393*	0.97 (0.82–1.15) *208*	0.79 (0.56–1.11) *36*	0.30
	Multivariable 1 *N of cases: 885*	0.91 (0.67–1.24) *45*	0.92 (0.77–1.09) *203*	1.00 *393*	1.00 (0.84–1.18) *208*	0.79 (0.56–1.12) *36*	0.63
	Multivariable 2 *N of cases: 885*	0.90 (0.66–1.23) *45*	0.91 (0.77–1.08) *203*	1.00 *393*	1.00 (0.85–1.19) *208*	0.80 (0.56–1.12) *36*	0.76
Rectal	Age-adjusted *N of cases: 426*	1.22 (0.80–1.87) *24*	1.09 (0.85–1.39) *100*	1.00 *175*	1.01 (0.79–1.29) *99*	1.37 (0.92–2.04) *28*	0.87
	Multivariable 1 *N of cases: 426*	1.21 (0.78–1.86) *24*	1.08 (0.84–1.39) *100*	1.00 *175*	1.02 (0.79–1.30) *99*	1.37 (0.92–2.05) *28*	0.95
	Multivariable 2 *N of cases: 426*	1.18 (0.77–1.82) *24*	1.07 (0.83–1.37) *100*	1.00 *175*	1.03 (0.80–1.32) *99*	1.40 (0.94–2.10) *28*	0.87
Colorectal	Age-adjusted *N of cases: 1311*	1.05 (0.82–1.35) *69*	0.99 (0.86–1.13) *303*	1.00 *568*	0.98 (0.86–1.13) *307*	0.97 (0.75–1.26) *64*	0.34
	Multivariable 1 *N of cases: 1311*	1.00 (0.77–1.28) *69*	0.97 (0.84–1.11) *303*	1.00 *568*	1.00 (0.87–1.15) *307*	0.97 (0.75–1.26) *64*	0.67
	Multivariable 2 *N of cases: 1311*	0.98 (0.76–1.26) *69*	0.96 (0.83–1.10) *303*	1.00 *568*	1.01 (0.88–1.16) *307*	0.98 (0.76–1.28) *64*	0.88

Multivariable 1 = adjusted for age, height, body mass index, duration of education, alcohol consumption, and smoking status

Multivariable 2 = additionally adjusted for intake of red meat, processed meat, dietary calcium, and dietary fibre

In multivariable repeated PA measurement analyses, after adjustment for repeated measurements of BMI and smoking status, the corresponding risks obtained were similarly not statistically significant (colon: HR = 0.82, 95% CI 0.58–1.16, *p*-trend = 0.27; rectal: HR = 1.40, 95% CI 0.93–2.09, *p*-trend = 0.74) (Table 3).

**Table 3:** Hazard ratios (95% CI) of colon, rectal, and colorectal cancers by physical activity level at baseline and follow-up (*n* = 79,184) in the NOWAC Study

		Physical activity level at baseline/follow-up					
Cancer	Models	1–2	3–4	5–6	7–8	9–10	*p* trend
Colon	Age-adjusted *N of cases = 833*	0.85 (0.61–1.19) *37*	0.96 (0.81–1.14) *199*	1.00 *379*	**0.81 (0.68–0.97)** *182*	0.83 (0.59–1.17) *36*	0.10
	Multivariable 1 *N of cases = 818*	0.79 (0.56–1.11) *36*	0.94 (0.79–1.12) *196*	1.00 *371*	0.84 (0.70–1.00) *180*	0.81 (0.57–1.15) *35*	0.23
	Multivariable 2 *N of cases = 818*	0.78 (0.55–1.10) *36*	0.94 (0.79–1.12) *196*	1.00 *371*	0.84 (0.70–1.01) *180*	0.82 (0.58–1.16) *35*	0.27
Rectal	Age-adjusted *N of cases = 398*	1.22 (0.79–1.88) *23*	0.90 (0.69–1.17) *82*	1.00 *173*	0.87 (0.67–1.12) *92*	1.36 (0.92–2.03) *28*	0.85
	Multivariable 1 *N of cases = 390*	1.24 (0.80–1.93) *23*	0.90 (0.68–1.17) *80*	1.00 *170*	0.85 (0.66–1.10) *89*	1.37 (0.91–2.04) *28*	0.90
	Multivariable 2 *N of cases = 390*	1.22 (0.78–1.89) *23*	0.88 (0.67–1.15) *80*	1.00 *170*	0.86 (0.66–1.11) *89*	1.40 (0.93–2.09) *28*	0.74
Colorectal	Age-adjusted *N ofcases = 1231*	0.96 (0.74–1.26) *60*	0.94 (0.81–1.09) *281*	1.00 *552*	**0.83 (0.72–0.96)** *269*	1.00 (0.78–1.30) *63*	0.22
	Multivariable 1 *N ofcases = 1208*	0.92 (0.70–1.21) *59*	0.93 (0.80–1.08) *276*	1.00 *541*	**0.84 (0.73–0.98)** *269*	0.99 (0.76–1.29) *63*	0.36
	Multivariable 2 *N ofcases = 1208*	0.91 (0.69–1.19) *59*	0.92 (0.80–1.09) *276*	1.00 *541*	**0.85 (0.73–0.98)** *269*	1.00 (0.77–1.30) *63*	0.47

Multivariable 1 = adjusted for age, height, body mass index, duration of education, alcohol consumption, and smoking status

Multivariable 2 = additionally adjusted for intake of red meat, processed meat, dietary calcium, and dietary fibre

Confidence intervals in bold have *p*-values less than 0.05

In analyses of the influence of changes in PA level on the risk of CRC, a statistically significant reduction in the risk of colon cancer was observed in those with “increased PA” when compared to those who remained “consistently moderately active” (HR = 0.69, 95% CI 0.50–0.95). We did not observe any significant association between women who were “consistently active”, “consistently inactive”, or those with “decreased PA” when compared to women who were “consistently moderately active” (Table 4).

**Table 4:** Hazard ratios (95% CI) of colon, rectal and colorectal cancers by *changes* in physical activity level between enrollment and follow-up (n = 44,498) in the NOWAC Study

		Changes in physical activity level				
Cancer	Models	Consistently active (PA 7–10) *[n = 9417]*	Consistently moderately active (PA 5–6) *[n = 13,189]*	Increased PA *[n = 7869]*	Decreased PA *[n = 6317]*	Consistently inactive (PA 1–4) *[n = 7706]*
Colon	Age-adjusted *N of cases: 393*	0.83 (0.62–1.11) *69*	1.00 *134*	**0.70 (0.51–0.97)** *51*	0.85 (0.62–1.16) *58*	0.91 (0.69–1.20) *81*
	Multivariable 1 *N of cases: 393*	0.86 (0.64–1.15) *69*	1.00 *134*	**0.69 (0.50–0.96)** *51*	0.82 (0.60–1.11) *58*	0.86 (0.65–1.14) *81*
	Multivariable 2 *N of cases: 393*	0.87 (0.65–1.16) *69*	1.00 *134*	**0.69 (0.50–0.95)** *51*	0.81 (0.60–1.11) *58*	0.86 (0.65–1.14) 81
Rectal	Age-adjusted *N of cases: 168*	**1.57 (1.03–2.41)** *43*	1.00 *42*	1.36 (0.86–2.16) *32*	1.16 (0.70–1.91) *24*	1.02 (0.63–1.66) *27*
	Multivariable 1 *N of cases: 168*	**1.54 (1.01–2.37)** *43*	1.00 *42*	1.34 (0.84–2.12) *32*	1.11 (0.67–1.84) *24*	1.02 (0.62–1.66) *27*
	Multivariable 2 *N of cases: 168*	**1.57 (1.02–2.42)** *43*	1.00 *42*	1.32 (0.83–2.10) *32*	1.11 (0.67–2.84) *24*	1.00 (0.61–1.63) *27*
Colorectal	Age-adjusted *N of cases: 561*	1.01 (0.80–1.28) *112*	1.00 *176*	0.86 (0.67–1.12) *83*	0.92 (0.71–1.20) *82*	0.94 (0.74–1.20) *108*
	Multivariable 1 *N of cases: 561*	1.03 (0.81–1.31) *112*	1.00 *176*	0.85 (0.65–1.10) *83*	0.89 (0.68–1.15) *82*	0.90 (0.71–1.15) *108*
	Multivariable 2 *N of cases: 561*	1.04 (0.82–1.32) *112*	1.00 *176*	0.84 (0.65–1.10) *83*	0.88 (0.68–1.15) *82*	0.90 (0.70–1.15) *108*

Multivariable 1 = adjusted for age, height, body mass index, duration of education, alcohol consumption, and smoking status

Multivariable 2 = additionally adjusted for intake of red meat, processed meat, dietary calcium, and dietary fibre

Confidence intervals in bold have *p*-values less than 0.05

Intriguingly, those who were “consistently active” were at an increased risk of rectal cancer when compared to women who were “consistently moderately active” (HR = 1.57, 95% CI 1.02–2.42) (Table 4).

## Discussion

In this nationally representative prospective study of Norwegian women, we did not find an association between PA level and the risk of CRC. These findings remained the same regardless of whether we used baseline data or repeated measurements, and after adjusting for known CRC risk factors. We also examined the influence of change in PA level on the risk of CRC and found that those who *increased* their PA from baseline to follow-up had a lower risk of colon cancer.

There is an established inverse relationship between PA and the risk of CRC, and several plausible explanatory biological mechanisms and hypotheses have been proposed [37, 38]. These mechanisms are not completely clear, however, the existing plausible hypotheses include the involvement of PA in the reduction of intestinal fecal transit time; increase production of motility-inducing prostaglandin F2α; alterations in sex hormones; reduction in insulin resistance and hyperinsulineamia; improved immune function; changes in free radical generation; and changes in body fat [37, 38]. There could be sex-specific differences in the physiological responses in some of these mechanisms that may place women at a disadvantage, or PA may also interact with other sex-specific factors influencing the responses [28, 29]. The Continuous Update Project on CRC by the WCRF/AICR recently inferred that PA of all types reduces the risk of CRC [9]. However, most of the epidemiological studies that corroborate this relationship have been conducted in men [11]. Results of studies in women have been largely inconsistent and less conclusive [10, 11, 14, 24].

As the exposure of interest, PA may be an intricate and difficult parameter to measure, especially in population-based studies. Inconsistencies may be associated with variations in PA instruments (assessment methods), the use of different domains of PA (occupational, household, transport, and recreational) with the frequency, duration, and intensity of PA in the investigation of the relationship. Nevertheless, the same heterogeneity in the assessment of PA in women also exist in the studies of the PA-CRC relationship in men; whereas the findings in men have been more consistent and largely conclusive [11, 13, 14, 24].

Our findings of no association between PA and the risk of CRC in women may be an accurate reflection of a true lack of association, which is consistent with findings from many previous prospective studies among women [10–22]. From the available prospective studies that included only women or gave sex-specific results, we identified 21 studies [6–8, 10–26, 39]. Thirteen of these studies found no association between PA and risk of CRC [10–22], six observed a statistically significant association [6–8, 23–25], while two reported both [26, 39]. The last two studies further underscore the discrepancies in the findings of PA-CRC relationship in women [26, 39].

Out of the 13 prospective studies that found no association, none of them used the same PA instrument we used in our study. Nevertheless, since our PA scale corresponds to *total* PA, including all the domains in one global score, we can compare our study to others that utilized *total* PA. For example, the questionnaire used in the National Institutes of Health-American Association of Retired Persons Diet and Health (NIH-AARP Diet and Health) Study [11] assessed participants’ detailed routine throughout the day, at home and work (daily routine activity), and sporting activities. Daily routine activity and sporting activity were analysed separately and neither were statistically significant (HR = 0.84, 95%CI 0.50–1.42, *p*-trend = 0.714 and HR = 0.87, 95%CI 0.71–1.06, *p*-trend = 0.536, respectively) in women. Interestingly, the same analyses were statistically significant in the participating men (HR = 0.86, 95%CI 0.66–1.12, *p*-trend = 0.007 and HR = 0.82, 95%CI 0.71–0.95, *p*-trend = 0.013, respectively). The Japan Public Health Center-based Prospective Study also found no relationship between *total* daily PA and CRC in women (HR = 0.82, 95%CI 0.56–1.21, *p*-trend = 0.198 for colon cancer; HR = 1.79, 95%CI 0.99–3.23, *p*-trend = 0.077 for rectal cancer) [20]. Corresponding analyses in the participating men from that study were statistically significant for colon cancer (HR = 0.58, 95%CI 0.48–0.79, *p*-trend < 0.001), but not for rectal cancer (HR = 0.88, 95%CI 0.57–1.36, *p*-trend = 0.464). The Framingham Study used the summary PA index of daily activity, which also relates to *total* daily PA. The authors observed no association between total daily PA and large bowel cancer (*p*-trend 0.89) among women, but they did report an association among men (*p*-trend 0.06) [18]. Likewise, the Breast Cancer Detection Demonstration Project (BCDDP), which used a PA instrument similar to that of Framingham Study, observed no association between *total* PA and the risk of colon cancer (HR = 1.15, 95%CI 0.76–1.75, *p*-trend = 0.77) [10].

The other nine prospective studies, which found no association between PA and CRC in women used various PA instruments and assessed different domains of PA. These ranged from recreational and non-recreational, with HR = 1.60, 95%CI 0.70–3.50 (inactivity-CRC relation) [17]; recreational and occupational, with HR = 0.86, 95%CI 0.77–1.03 [12]; recreational only, with HR = 0.77, 95%CI 0.43–1.38, *p*-trend = 0.27 [14], HR = 0.90, 95%CI 0.56–1.46, *p*-trend = 0.68 [15], HR = 0.89, 95%CI 0.50–1.60 [16], HR = 0.95, 95%CI 0.68–1.39, *p*-trend = 0.75 [22]; non-recreational only, with HR = 0.94, 95%CI 0.40–2.21 [21], amount of time spent walking, with HR = 1.02, 95%CI 0.60–1.75, *p*-trend = 0.91 [19]; to metabolic equivalent (MET) hours per day, with HR = 1.16, 95%CI 0.76–1.77, *p*-trend = 0.569 [13]. However, some of these studies observed statistically significant associations among men from the same studies [13, 14, 16, 19].

On the other hand, six prospective studies reported a significant association between PA and colon cancer or CRC [6–8, 23–25]. The Nurses’ Health Study found significant inverse association between recreational PA and incidence of colon cancer in women (HR = 0.54, 95%CI 0.33–0.90, *p*-trend = 0.03) consistent with results found in men [6]. The Nord-Trøndelag Health Study conducted in Norway also found a significant association among women who reported high recreational PA versus no PA (HR = 0.77, 95% CI 0.53–0.98, *p*-trend = 0.03). No linear association was found for rectal cancer risk (*p*-trend = 0.74) [7]. Another population-based cohort study in women in Norway found recreational PA to be associated with decreased risk of colon cancer (HR = 0.62, 95% CI 0.40–0.97, *p*-trend = 0.25) [8]. However, The California Teachers Study found that lifetime recreational PA reduces colon cancer risk among postmenopausal women who had never taken hormone therapy (HR = 0.51, 95% CI 0.31–0.85, *p*-trend = 0.02), but not in postmenopausal women with history of hormone therapy use (HR = 0.98, 95% CI 0.66–1.44 *p*-trend = 0.49) [23]. One thing is conspicuously common to these studies: they all utilized the single domains of either recreational [6–8, 23] or occupational [8, 24, 25] PA. This may have effectively excluded the household (domestic or family care) PA domain, which is mostly important for the female population [27]. This could partly account for the gender bias in the appraisal of PA in epidemiological studies [40]. On the other hand, it may be relatively easy to remember and thus *simpler* to appraise recreational and occupational PA compared to *total* PA.

According to our findings, those who *increased* their PA from baseline to follow-up had a lower risk of colon cancer, thus this lower risk may very well be a marker of a generally healthy lifestyle. However, we found no association between those who were *consistently* active and the risk of colon cancer. This further portrays that both short and consistent PA over a period of time may not confer protection against colon cancer in women. The association between long-term PA and a reduced risk of colon cancer (consistently active vs consistently inactive) is more often seen in men [39, 41], and even then it is inconsistent [42]. Intriguingly, women who were *consistently* active were at an increased risk of rectal cancer when compared to those who were *consistently* moderately active. This result must be interpreted with caution as it could be a spurious finding, which is probably due to another associated factor. This is because the finding on its own has no plausible physio-biological explanations.

The present study has some limitations. Our PA measurement may not have been sensitive enough to detect perhaps small effect of PA on CRC among women. The PA level in our study was self-reported through questionnaires and thus is inevitably susceptible to measurement error [43]. Unfortunately, in large population-based studies, one may not be able to use more accurate PA assessment methods, such as the accelerometer and gyroscope. Furthermore, although the PA assessment used in our study gave a *total* PA score, this score lacks quantification and distinguishability of the domains involved, the frequencies, durations, and intensities of the PA [32]. The ordinal scale measures self-perceived PA, which is subjected to individual frame of reference, which may differ widely [28]. Thus, one should be cautious of this limitation while interpreting the results. Notwithstanding, the PA instrument we used has been validated, and the results show that the scale is sufficient to differentiate between levels of the total amount of PA. The Spearman correlation coefficient was found to be moderate at 0.36–0.46 with *p*-value less than 0.001 [32]. This compares well with the International Physical Activity Questionnaire, which reported criterion validity by Spearman correlation of a median of 0.30 in a validation study across 12 countries [44]. The covariates in our study were also self-reported and are therefore prone to the errors inherent to self-reporting. Indeed, self-reporting leads to a tendency for people to overstate desirable behaviours, such as PA, dietary habits, and alcohol consumption habits, thereby introducing some level of misclassification error [45]. We used only one measure of the dietary intakes, taken at enrollment. These intakes likely change over time and may be invalid over the length of the study period [46]; thus, residual confounding cannot be excluded. Nevertheless, the information in the NOWAC Study on PA, BMI, dietary habits, and alcohol consumption habits have been validated with satisfactory results [32, 47–49]. The self-reported duration of education has been compared to the relevant national registries and no statistical differences were found [30]. Accordingly, this self-reporting method is judged to be adequate and pragmatic, especially considering the large sample size of the NOWAC Study. Our study lacked information on family history of CRC. Women who have a familial predisposition to developing CRC may be more health conscious than others, which may cause residual confounding. Likewise, we lacked information on use of aspirin and other non-steroidal anti-inflammatory drugs (NSAIDs) by our participants. Regular use of aspirin and other NSAIDs are suggestive of protection against colon adenoma and cancer [50]. This may also be a source of confounding.

Our study has several strengths. These include the prospective and population-based design, the large sample size, the long follow-up time, information on important confounding factors, and the use of a high-quality national cancer registry to identify cases of CRC [31]. The NOWAC cohort consists of participants who were randomly recruited from the general population and is representative of the Norwegian female population aged 30 to 70 years [32]. The external validity of the NOWAC cohort has been found to be acceptable [30]. We used repeated measurements of PA level, BMI, and smoking status in order to account for changes in these variables over time and to attenuate the risk of measurement error. The availability of data on PA level at two different time points also allowed us to investigate changes in PA levels, which is a vital strength of this study. The self-reported BMI and the food frequency questionnaire in the NOWAC Study have been validated [47–49]. There is a substantial agreement between the self-reported and measured BMI values [49], while 24-h dietary recall studies found the food frequency questionnaire to be reliable [47, 48].

## Conclusions

Our data do not support the hypothesis that *total* physical activity, nor consistent participation in PA over a period of time, is associated with a reduced risk of CRC in women. Thus, women may need to look beyond PA in order to reduce their risk of CRC.

## Abbreviations

BMI: body mass index
CI: confidence interval
CRC: colorectal cancer
CUP: Continuous Update Project
HR: hazard ratio
ICD-10: International Statistical Classification of Diseases, Injuries and Causes of Death 10th revision
NOWAC: the Norwegian Women and Cancer Study;
PA: physical activity
WCRF/ AICR: World Cancer Research Fund/American Institute for Cancer Research
